# Evaluation of laparoscopic surgery effects on pain severity and quality of life in different subtypes of endometriosis: A follow-up study

**DOI:** 10.18502/ijrm.v23i2.18494

**Published:** 2025-05-01

**Authors:** Mania Kaveh, Haniye Malakouti, Shahla Chaichian, Abolfazl Mehdizadeh Kashi, Mahdi Afshari, Kambiz Sadegi

**Affiliations:** ^1^Department of Obstetrics and Gynecology, School of Medicine, Amir Al Momenin Hospital, Zabol University of Medical Sciences, Zabol, Iran.; ^2^Iranian Scientific Society of Minimally Invasive Gynecology, Tehran, Iran.; ^3^School of Medicine, Zabol University of Medical Sciences, Zabol, Iran.; ^4^Endometriosis Research Center, Iran University of Medical Sciences, Tehran, Iran.; ^5^Department of Obstetrics and Gynecology, School of Medicine, Endometriosis Research Center, Hazrat-e Rasool General Hospital, Iran University of Medical Sciences, Tehran, Iran.; ^6^Department of Community Medicine, School of Medicine, Pediatric Gastroenterology and Hepatology Research Center, Zabol University of Medical Sciences, Zabol, Iran.; ^7^Department of Anesthesiology, School of Medicine, Amir Al Momenin Hospital, Zabol University of Medical Sciences, Zabol, Iran.

**Keywords:** Endometriosis, Laparoscopy, Pain, Quality of Life.

## Abstract

**Background:**

Studies have shown that endometriosis significantly has a negative impact on women's mental health and quality of life (QoL), resulting in these participants experiencing a diminished QoL.

**Objective:**

This study aimed to evaluate the effect of laparoscopic surgery on the severity of pain and the QoL of women with different types of endometriosis.

**Materials and Methods:**

In this follow-up study 50 women with endometriosis, who underwent laparoscopic surgery, who met a high visual analog score ( > 6) with impaired QoL, lack of response to analgesics, infertility with pain unresponsive to assisted reproductive therapy, and involvement of other organs such as the bowel or ureter at the Amir Al Momenin hospital, Zabol, Iran, from August 2022 to January 2023 were enrolled. The participants were categorized into 2 groups: those with stage IV endometriosis and those with lower stages (I-III). Groups according to the higher occurrence of grade 4 endometriosis, as 55.1% were affected by it. The pain score was measured using the visual analog score, and the participants' QoL score was measured using the endometriosis health profile questionnaire.

**Results:**

The mean age of participants was 32 yr, with a standard deviation of 8.6 yr. Participants were divided into 2 groups: those with grade 4 endometriosis (55.1%) and those with a grade lower than 4, as classified by the American Society for Reproductive Medicine. Over 12 months, both groups experienced a significant decrease in pain severity and QoL scores (p < 0.0001). However, the changes in pain and QoL scores between the 2 groups were not statistically significant (p = 0.520 and p = 0.984, respectively).

**Conclusion:**

Laparoscopic treatment can reduce pain and QoL scores (increase the QoL indices) in women with endometriosis, regardless of the disease's severity.

## 1. Introduction 

The condition known as endometriosis is identified by the presence of endometrial glands and stroma outside the endometrial cavity and uterine muscles. Endometriosis tissues are most commonly found in the pelvic region; however, they have the potential to develop in nearly any part of the body. Pelvic endometriosis lesions can be classified into subtypes: superficial, deep, and endometrioma cysts. These cysts contain both blood and endometrial tissues (1–3). The appearance and size of subtypes of endometriosis are different. In addition to endometrial glands and stroma, these lesions contain fibrous tissue, blood, and cysts. Endometriosis is associated with an increased risk of adverse pregnancy outcomes, epithelial ovarian cancer, and atherosclerosis. This disease can be associated with many uncomfortable and debilitating symptoms, but some participants may also be asymptomatic (4–6).

Endometriosis is classified into 4 stages: I (minimal), II (mild), III (moderate), and IV (severe), depending on the location, spread, and depth of the implants (7). The prevalence of endometriosis within the general population remains uncertain, as estimates vary widely based on the characteristics of the study population (e.g., symptomatic versus asymptomatic individuals) and the diagnostic approach utilized (clinical evaluation or surgical confirmation). This disease has been reported in up to 70% of women with chronic pelvic pain (8). If the diagnosis is confirmed during laparoscopy, tissue resection should be performed.

However, a comprehensive evaluation of untreated endometriosis in adolescents still needs to be improved in terms of long-term follow up. Moreover, more data are needed regarding the efficacy of laparoscopic treatment for diverse types of endometriosis (9).

This study investigates the quality of life (QoL) of endometriosis participants after endometriosis correction surgery according to different subtypes of the disease. Accordingly, the present study aims to evaluate the effect of laparoscopic endometriosis surgery on pain severity and QoL in different subtypes of endometriosis.

## 2. Materials and Methods

This follow-up study was conducted on 50 women who underwent laparoscopy with a diagnosis of endometriosis at the Amir Al Momenin hospital, Zabol, Iran, from August 2022 to January 2023. The researchers collected the information of all the eligible participants.

Inclusion criteria: surgically confirmed endometriosis, candidates for laparoscopic surgery due to severe pain (visual analog score [VAS] > 6) despite conservative treatment and due to the involvement of vital organs, written informed consent to undergo laparoscopic surgery and participate in the study.

Exclusion criteria: lack of participate consent, diagnosis of adenomyosis or uterine fibroids, diagnosis of irritable bowel syndrome or inflammatory bowel disease.

All women with endometriosis who had the following conditions underwent laparoscopy: high score of the VAS ( > 6) with impaired QoL, no response to analgesics, presence of infertility with pain and without response to assisted reproductive therapy, involvements of other organs with endometriosis like bowel or ureter involvement (10). Then, confirmed endometrioses were resected by surgical procedure -an expert gynecologist operated on all participants.

Laparoscopic surgery is a corrective procedure for endometriosis, performed based on the areas involved.

The initial approach involves retroperitoneal dissection, followed by ureteral mobilization, and, if necessary, dissection of the pararectal and paravesical spaces, along with excision of the posterior cul-de-sac. In cases where endometriosis implants are identified, they are excised, and if an endometrioma cyst is detected, it is also removed. During the excision process, special attention is given to maintaining anti-Müllerian hormone (AMH) levels through meticulous and targeted hemostasis. Electrosurgery is minimized to avoid damage, and suturing is used for hemostasis. Special attention is paid to ovarian reserve AMH to ensure minimal damage to ovarian tissue, which is sutured at the end.

Verogest tablets were administered for up to 18 months to participants who did not wish to conceive.

Additionally, all participants had their AMH levels evaluated. After consulting with the infertility service, participants with an AMH level > 2.5, who suffered from severe pain unresponsive to medical treatments, or those with very large or progressively increasing cysts were regarded as surgical emergencies for endometriosis, regardless of their age. For participants experiencing infertility, the infertility service assessed the need for oocyte or embryo preservation, which was performed prior to the surgical procedure. Notably, surgical indications at the age of 18 can include any of the following: severe pelvic pain with a high VAS score that has not responded to medical treatments, severe endometriosis involvement causing bowel obstruction, ureteral involvement, or a large cyst size.

Participants were classified into 2 groups based on the severity of endometriosis, as assessed intraoperatively using the American Society for Reproductive Medicine (ASRM) classification (11). The grouping was determined during surgery, not retrospectively based on pain severity.

According to the ASRM classification, endometriosis staging is as follows:

1. Stage I (minimal): < 15 points

2. Stage II (mild): 16–40 points

3. Stage III (moderate): 41–100 points

4. Stage IV (severe): > 100 points

In this study, participants were divided into 2 groups: those with stage IV endometriosis and those with lower stages (I-III). The classification was based on surgical findings, not pain severity.

The pain score of the participants was measured based on the VAS, which is a tool for measuring pain intensity by the participants between 0 (no pain) and 10 (most pain imaginable) (12). The endometriosis health profile (EHP-30) questionnaire assessed the participants' QoL score. This questionnaire consists of 30 questions covering 5 areas: pain, degree of control, emotions, social support, and self-perception. Participants responded using a 5-point likert scale. Nojomi et al. assessed the reliability and validity of the Persian version of the EHP-30 questionnaire (13). All participants were followed up for 12 months after surgery. Pain severity and QoL were measured before the operation, 3, 6, and 12 months after the laparoscopic resection. For this purpose, participants were invited to the gynecology clinic during these times. After assessing all clinical and paraclinical factors, as well as the potential adverse outcomes of the surgery, participants underwent in-person interviews conducted by a skilled researcher utilizing the abovementioned instruments. The researcher evaluated the pain severity level and assessed the QoL score. In case of non-adherence, the gynecologist contacted the participants or their families and encouraged them to visit. If participants could not participate in the visit or refused to be referred to the clinic, the required information was collected by phone contact.

### Sample size

The required sample size was estimated as 50 subjects to detect 67% relief in symptoms based on the results of the study performed by Abbott et al. (14), considering the maximum 13% as the alpha error using OpenEpi statistical software based on the following formula (15): 


$$n=\frac{Z^{2}P\left(1-P\right)}{d^{2}}=50$$

n refers to the estimated sample size, p stands for the hypothesized frequency based on the previous literature, d is the absolute precision or maximum marginal alpha error, and Z is a statistic corresponding to the confidence level (1.96 for 95% confidence interval).

### Ethical Considerations

The Ethics Committee of Zabol Medical Sciences University, Zabol, Iran, approved this study (Code: IR.ZBMU.REC.1401.045). Prior to the study, participants provided verbal consent after being fully informed, and strict confidentiality was maintained regarding all participant information.

### Statistical Analysis

Continuous data were described by mean ± SD, and categorical data were described by percent frequency. In groups with each endometriosis grade, the mean pain severity score and QoL from baseline to 12 months after surgery were compared using a paired *t* test or Wilcoxon test. The pain and QoL of participants with different subtypes during the follow-ups were compared with ANOVA models, considering baseline values as a covariate and grade of disease as a factor. All statistical analyses were performed using Statistical Package for the Social Sciences software (SPSS, IBM, Inc., Armonk, NY), version 27. A p ≤ 0.05 was considered significant.

## 3. Results

In this study, 50 women with endometriosis who underwent laparoscopic surgery were studied. The mean age of women was 32 ± 8.6 yr. The youngest and oldest were 18 and 53 yr old, respectively. The highest grade of endometriosis was grade 4 (56%), 15 participants (30%) had grade 3, 6 participants (12%) had grade 2, and one participant (2%) had grade 1 endometriosis. Due to the higher population of participants with grade 4, the researchers divided all participants into 2 groups: those with grade 4 endometriosis and those with grades < 4, based on the ASRM classification. Women with endometriosis grades < 4 had a mean age of 34.9 ± 9.8 yr, and those with grade 4 had a mean age of 30.3 ± 7.09 yr. No significant difference was observed between the 2 groups (p = 0.065).

Table I compared the pain severity between the 2 groups based on grades during follow up. Table II indicates the evaluation of the women's QoL in the 2 groups during follow up.

**Table 1:** Comparison of pain severity scores in groups during treatment

**Endometriosis grade**	**Pain severity score**				P-value*	P-value**
	**Pre-surgery**	**After 3 months**	**After 6 months**	**After 12 months**		
**> 4**	8.95 ± 0.09 9 (9–10)	4.40 ± 3.01 4 (1–7)	3.50 ± 2.90 2 (1–6)	2.68 ± 2.74 1 (1–3)	< 0.0001	0.520
**4**	8.48 ± 1.67 9 (8.25–9)	4.29 ± 2.65 4.5 (1.25–7)	3.0 ± 2.27 2 (1–5)	2.33 ± 2.14 1 (1–3)	< 0.0001	
**P-value**	0.244	0.890	0.502	0.621	—	
Data presented as Mean ± SD (median, interquartile range), ANOVA, *Comparison of pain severity during 12 months, **Comparison of pain severity between groups						

**Table 2:** Comparison of QoL scores in the groups during follow up

**Endometriosis grade**	**QoL score**				P-value*	P-value**
	**Pre-surgery**	**After 3 months**	**After 6 months**	**After 12 months**		
**> 4**	54.59 ± 37.08 55 (26.5–80.75)	51.18 ± 36.77 51 (20.25–70.75)	38.09 ± 33.37 33.5 (9.75–60.25)	30.31 ± 30.55 22.5 (9.75–60.25)	< 0.0001	0.984
**4**	59.44 ± 35.01 62.5 (34–85.75)	54.66 ± 34.63 53 (27.5–79.5)	35.0 ± 30.73 34 (3–56)	25.77 ± 28.59 21.5 (0–56)	< 0.0001	
**P-value**	0.641	0.735	0.738	0.594	—	—
Data presented as Mean ± SD (median, interquartile range), ANCOVA, *Comparison of QOL during 12 months, **Comparison of QOL between groups. QoL: Quality of life						

## 4. Discussion 

This study showed that laparoscopic surgery in women with endometriosis during the 12 months of follow-up reduced the pain severity and increased participants' QoL. These effects did not differ between different types or different grades of endometriosis. Chronic pelvic pain, impaired daily movement, and decreased QoL are the effects of endometriosis on participants' lives (16).

One study evaluated the effects of laparoscopy on QoL and pain relief in participants with endometriosis and found that pain scores significantly decreased after laparoscopy. Furthermore, the researchers observed a significant improvement in the participants' activity, well-being, sexual relationship, and sexual activity following laparoscopic surgery. The study's findings indicated that this surgical intervention effectively reduced pain and enhanced the overall well-being of individuals diagnosed with endometriosis (17). The current study found that laparoscopic surgery significantly reduced pain severity and increased QoL over 12 months. The effects of this method are not associated with the types of endometriosis. The EHP-30 questionnaire contains 5 subscales: pain, control and powerlessness, social support, emotional well-being, and self-image (18).

According to this research, individuals experienced enhanced control and powerlessness, increased social support, improved emotional well-being, and a positive self-image following laparoscopic surgery for endometriosis. Additionally, there was an improvement in their pain levels. Moreover, the present findings align with another study.

They demonstrated that the VAS score of the participants significantly decreased in the long term after laparoscopy compared to preoperative. The scores for dysparonychia, dysmenorrhea, dysuria, and chronic pelvic pain before surgery were 8.5, 6.6, 7.4, 5.6, and 4.1, respectively, and postoperative, they decreased to 2.9, 1.6, 2.4, 1.3, and 1.2, respectively. The results of this study indicated that laparoscopic management of deep endometriosis is a valid treatment to reduce pain and increase the QoL of participants (19). While this study did not specifically measure scores for dyspareunia, dysmenorrhea, and dysuria, its findings align with those of another study. This research discovered that laparoscopic surgery of endometriosis significantly improved pain and QoL scores.

Another study evaluated the pain and participants' QoL during 3 yr after laparoscopy due to endometriosis. They found that the pain and participants' QoL improved during 1 yr, and the obtained scores remained stable for 3 yr (20). Their study results were similar to those of the present study. The evaluation period is a significant difference between the 2 studies: 1 yr in the current study and 3 yr in the other. Another research found that after 1 yr, participants experienced significant improvements in both QoL and pain severity compared to their preoperation condition (21). Although this study did not discuss the disease grade, it revealed that participants who underwent complete excision were more satisfied than those with incomplete excision. Additionally, research by Laguerre et al. demonstrated that in a long-term follow-up (5 yr) of participants who had surgery for endometriosis, QoL improved significantly, and only 8.6% of participants required reoperation (22).

This study's findings indicated that performing laparoscopic surgery in women with endometriosis during the 12-month follow-up had increased the QoL index and women's satisfaction. However, the grade of endometriosis disease had no effect on the QoL and pain score in these participants after laparoscopy, and women who had high grades had similar outcomes to women with low-grade endometriosis. This study highlights a new finding: laparoscopic surgery for treating endometriosis significantly improves various QoL parameters, particularly alleviating pain. These results suggest that laparoscopic excision of endometriosis, with the preservation of the uterus, ought to be regarded as a feasible alternative during the treatment discussions for endometriosis (23). Nevertheless, further prospective studies with larger sample sizes are recommended to exclude the grading of the disease's grading from the indications for surgery.

### Strengths and limitations

The present study has certain limitations that need to be acknowledged when interpreting the findings. Primarily, the relatively small sample size and its restriction to a single center may limit the broader applicability and generalizability of the results.

Second, the follow-up period was only 12 months; a longer follow-up could provide more insights into the long-term effects of laparoscopic surgery on pain severity and QoL.

Moreover, for assessing the pain score and QoL in endometriosis participants, the classification of bowel involvement and adhesions can be considered. However, due to the unavailability of such data to the authors and the lack of consideration for these details in the initial study design, this can be regarded as a study limitation. Finally, surgical indications at the age of 18 may include any of the following: severe pelvic pain with a high VAS score that has not responded to medical treatments, severe endometriosis causing bowel obstruction, ureteral involvement, or large cyst size.

## 5. Conclusion

This study's findings indicate that laparoscopy can effectively reduce pain intensity and frequency while enhancing the QoL for women with endometriosis, regardless of the disease stage. Educating and raising awareness among those involved in this field is crucial.

##  Data Availability

The data supporting the findings of this study are available upon reasonable request from the corresponding author.

##  Author Contributions

M. Kaveh and K. Sadegi designed and supervised the study. H. Malakouti conducted the research. M. Afshari evaluated and analyzed the results of the study. Furthermore, Sh. Chaichian and A. Mehdizadeh Kashi reviewed the article. All the authors approved the final manuscript and took responsibility for the integrity of the data.

##  Conflict of Interest

The authors declare that there is no conflict of interest.
